# Towards the Development of a Low-Cost Device for the Detection of Explosives Vapors by Fluorescence Quenching of Conjugated Polymers in Solid Matrices

**DOI:** 10.3390/s17112532

**Published:** 2017-11-03

**Authors:** Liliana M. Martelo, Tiago F. Pimentel das Neves, João Figueiredo, Lino Marques, Alexander Fedorov, Ana Charas, Mário N. Berberan-Santos, Hugh D. Burrows

**Affiliations:** 1 Department of Chemistry, University of Coimbra, 3004-535 Coimbra, Portugal; burrows@ci.uc.pt; 2 Centro de Química-Física Molecular (CQFM) and the Institute of Nanoscience and Nanotechnology (IN), Instituto Superior Técnico, University of Lisbon, 1049-001 Lisbon, Portugal; berberan@tecnico.ulisboa.pt; 3 Institute of Systems and Robotics (ISR), University of Coimbra, 3030-290 Coimbra, Portugal; tiago.pimenteldasneves@epfl.ch (T.F.P.d.N.); jfigueiredo@assystem.com (J.F.); lino@isr.uc.pt (L.M.); aleksander@mail.ist.utl.pt (A.F.); 4 Instituto de Telecomunicações, Instituto Superior Técnico, Av. Rovisco Pais, 1049-001 Lisbon, Portugal; ana.charas@lx.it.pt

**Keywords:** conjugated polymers, explosives detection, trace analysis, optical sensor, luminescence sensor

## Abstract

Conjugated polymers (CPs) have proved to be promising chemosensory materials for detecting nitroaromatic explosives vapors, as they quickly convert a chemical interaction into an easily-measured high-sensitivity optical output. The nitroaromatic analytes are strongly electron-deficient, whereas the conjugated polymer sensing materials are electron-rich. As a result, the photoexcitation of the CP is followed by electron transfer to the nitroaromatic analyte, resulting in a quenching of the light-emission from the conjugated polymer. The best CP in our studies was found to be poly[(9,9-dioctylfluorenyl-2,7-diyl)-co-bithiophene] (F8T2). It is photostable, has a good absorption between 400 and 450 nm, and a strong and structured fluorescence around 550 nm. Our studies indicate up to 96% quenching of light-emission, accompanied by a marked decrease in the fluorescence lifetime, upon exposure of the films of F8T2 in ethyl cellulose to nitrobenzene (NB) and 1,3-dinitrobenzene (DNB) vapors at room temperature. The effects of the polymeric matrix, plasticizer, and temperature have been studied, and the morphology of films determined by scanning electron microscopy (SEM) and confocal fluorescence microscopy. We have used ink jet printing to produce sensor films containing both sensor element and a fluorescence reference. In addition, a high dynamic range, intensity-based fluorometer, using a laser diode and a filtered photodiode was developed for use with this system.

## 1. Introduction

Part of the extensive research in conjugated polymers (CPs) and conjugated polyelectrolytes (CPEs) is motivated by their capacity as sensitive fluorescent materials for chemo- and biosensing. They offer a broad range of possibilities for transforming analyte receptor interactions, as well as nonspecific interactions, into observable (transducible) responses [1,2]. Amplified quenching in fluorescent CP was introduced by Swager and Zhou [3] and opened the way for novel sensory materials using this important class of conjugated polymers. In 1998, Yang and co-workers [4] used a fluorescence quenching transduction mechanism together with the amplifying nature of conjugated polymers to develop a material highly sensitive to 2,4,6-trinitrotoluene (TNT) vapors, the major explosive component of landmines. One peculiarity of nitroaromatics which may be used in detection based on fluorescence techniques is their electron-accepting capability. CPs are promising for redox sensing because they are normally electron donors. This donor behavior is further enhanced in their delocalized pi excited states. This excited state delocalization is crucial because the resulting exciton migration along the polymer chain increases the frequency of interaction with a bound quencher, in this case the nitroaromatic analytes, which contributes to improve detection sensitivity. For these reasons, nitroaromatic analytes can efficiently quench the emission of CP by photoinduced electron transfer process. As a practical result, photoexcitation of the conjugated polymer is followed by electron transfer to the nitrated organic compounds, resulting in a quenching of the CP fluorescence. Fluorescence quenching sensing methods are promising for rapid and sensitive detection of explosives vapors, and possess major advantages, including high sensitivity signal output and operational simplicity [5].

The detection of explosives is a major quest for security in many civilian and military environments, and is usually carried out through the sensing of the vapor emitted by the explosives, or of markers present with them. These sensors must satisfy several criteria, such as sensitivity, reversibility and the capability for real-time signal processing. For nitroaromatic explosives, sensing of a few parts per billion or less of the analyte vapor is mandatory, and should be accompanied with rapid and, ideally, reversible changes in the sensor output. Some assessments of explosives containing soils have been performed, and it has been indicated that the concentration of TNT is around 10–100 ng/kg. The vapor concentration is even lower, around the 100 pg/kg to 100 fg/kg level [6]. For in-field detection of such materials, a portable system would be highly beneficial.

**Figure 1:**
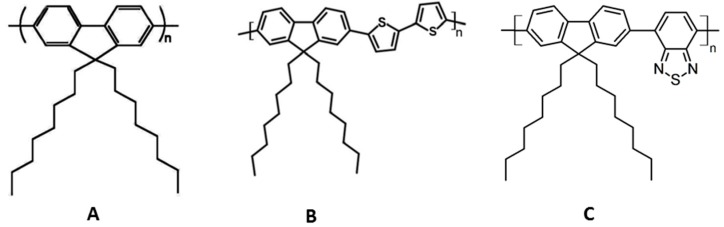
Structures of the CPs used: (**A**) poly[9,9-dioctylfluorene-2,7-diyl] (PFO); (**B**) poly[(9,9-dioctylfluorenyl-2,7-diyl)-co-bithiophene] (F8T2); and (**C**) poly[(9,9-dioctylfluorenyl-2,7-diyl)-alt-co-(1,4-benzo-2,10,3- thiadiazole)] (F8BT).

In order to address these issues, we have developed a new conjugated polymer-based optical sensor of trace explosives vapors. For the chemosensory material, we have used hairy-rod polymers [7], an important class of π-conjugated polymers, such as poly(fluorene-2,7-diyl)s (PFs). These have excellent photoluminescence quantum yields, good thermal stability, and good solubility in several solvents [8]. The linear side chain poly[9,9-dioctylfluorene-2,7-diyl] (PFO) and its homologue poly[9,9-dioctylfluorenyl-2,7-diyl)-co-bithiophene]) (F8T2) were used in this study (Figure 1). Detailed spectroscopic and photophysical properties of these polymers have been presented elsewhere [9].

For many practical applications, it is desirable to incorporate CPs in an appropriate porous inert matrix. In this work, we used ethyl cellulose (EC) to incorporate the CPs. Both CPs exhibit high sensitivity in ethyl cellulose films when exposed to nitrobenzene (NB) and 1,3-dinitrobenzene (DNB) vapors. These are chosen as models or markers of more common nitroaromatic explosives, such as TNT or RDX. EC is the most common insoluble cellulose derivative used and is available in a variety of viscosity grades, according to the molecular weight range of the products. The molecular weight affects the mechanical properties, which have fundamental importance for producing intact films, depending on the application [10]. Plasticizers are generally used to improve the mechanical properties of a polymer matrix. This occurs because the plasticizer can decrease the intramolecular forces between the polymer chains, reducing the glass transition temperature and increasing the permeability of the polymer matrix to gases or other analytes [11]. In this work, we use polyethylene glycol and polypropylene glycol with different molecular weights as plasticizers to improve the mechanical properties and permeability of our polymer matrices.

This contribution is divided in two parts. First, we report absorption, emission spectra, and fluorescence lifetimes of the PFO and F8T2 in ethyl cellulose films, the structural characterization of the thin films, and then discuss the ability of these materials to sense TNT model compounds. In the second part, we study the improved polymer matrices produced by the introduction of plasticizers which increase the sensitivity to TNT-like compounds when compared with the non-plasticized ones. We also develop other methods for CP device preparation in the solid matrix, such as ink jet printing technology: in this case we added an internal reference, a CP whose fluorescence is not quenched by the TNT-like molecules, to provide potential for ratiometric sensing. In this condition, we print different zones with the two CPs, and use as “paper” the non-plasticized ethyl cellulose matrix.

## 2. Materials and Methods

### 2.1. Materials

Ethyl cellulose of viscosity grade 100 cP, was acquired from Sigma-Aldrich (St. Louis, MO, USA) and used without any treatment. The conjugated polymers, poly[9,9-dioctylfluorene-2,7-diyl] (PFO, Mw ≥ 20,000) poly[9,9-dioctylfluorenyl-2,7-diyl)-co-bithiophene]) (F8T2, Mn > 20,000) and poly(9,9-dioctylfluorene-alt-benzothiadiazole) (F8BT, Mn ± 17,000-23,000) were from Sigma-Aldrich. Solutions for film preparation were made by dissolving ethyl cellulose and the CPs (200–500 ppm) in toluene (GPS grade, Carlo Erba Reagents) at room temperature. Nitrobenzene (ACS reagent, 99%) and 1,3-dinitrobenzene (99%) were from Sigma-Aldrich.

### 2.2. Film Preparation and Ethyl Cellulose Plasticization

Films were prepared by solution casting of a mixture of ethyl cellulose and the CPs (200–500 ppm) from toluene at room temperature. To ensure good optical quality of the films, the solvent was evaporated slowly at room temperature (72 h) and the last traces removed in an oven at 60 °C for 10 min. No differences in fluorescence behavior were observed when samples were left at this temperature for longer times. Plasticized films were obtained by the addition of 1–10 wt % of polyethylene glycol (600 and 3400) and polypropylene glycol (average Mw = 1000) to EC, followed by its dissolution in the solvent. The thicknesses of the films were 400–600 µm as measured with a micrometer (Etalon Rolle, Switzerland).

### 2.3. Ink Jet Printing

A FUJIFILM Dimatix Materials printer DMP-2800 Series was used for printing films. This is suitable for printing the CPs on an appropriate matrix. Toluene solutions of the CPs were used to fill disposable cartridges that have 16 individually-tunable, piezo-actuated nozzles. Cartridges are available for dispensing 10 pL or 1 pL drops. Drops were printed by voltage-driven deformations of a membrane wall of a chamber behind each nozzle. The segments of this action make up a waveform that is optimized for each ink, as well as the intended print job.

### 2.4. Luminescence Characterization

The UV spectroscopic measurements were performed at room temperature with a Shimadzu UV-3101PC UV-VIS-NIR spectrometer, using cells of 1.0 cm optical path length for solution measurements. The emission and excitation spectra were recorded with a Horiba Jobin-Ivon SPEX Fluorolog 3−22 fluorescence spectrometer. The Fluorolog consists of a modular spectrofluorimeter with double-grating monochromators for excitation (200–950 nm range, optimized in the UV with a blaze angle at 330 nm) and emission (200–950 nm range, optimized in the visible and with a blaze angle at 500 nm). The bandpass for excitation and emission was 5 nm with a wavelength accuracy of ± 0.5 nm. The excitation source consisted of an ozone-free 450 W xenon lamp. The emission detector employed was a Hamamatsu R928 photomultiplier, with a photodiode as the reference detector. The fluorescence quenching of the film was measured in a sealed cuvette containing the nitrobenzene or 1,3-dinitrobenzene vapors at room temperature (293 K).

Time-resolved picosecond fluorescence intensity decays were obtained by the single-photon timing method with laser excitation, with the set-up described elsewhere [12]. Decay data analysis with a sum of exponentials was achieved by means of a Microsoft Excel spreadsheet specially designed for lifetime analysis that considers deconvolution with the instrument response function (IRF) [13].

### 2.5. Structural Characterization

A confocal laser scanning microscope (Leica TCS-SP5) equipped with a CW Ar ion laser (458, 465, 488, 496, and 514 nm) and a pulsed Ti:sapphire (Spectra-Physics Mai Tai BB, 710–990 nm, 100 fs, 80 MHz) was used to obtain images of the films.

SEM was performed with a Hitachi S2400 microscope and the images were recorded by software Quantax (Bruker; Billerica, MA, USA). Samples were coated with gold and registered at 50× and 500× magnification.

### 2.6. Sensor Prototype

**Figure 2:**
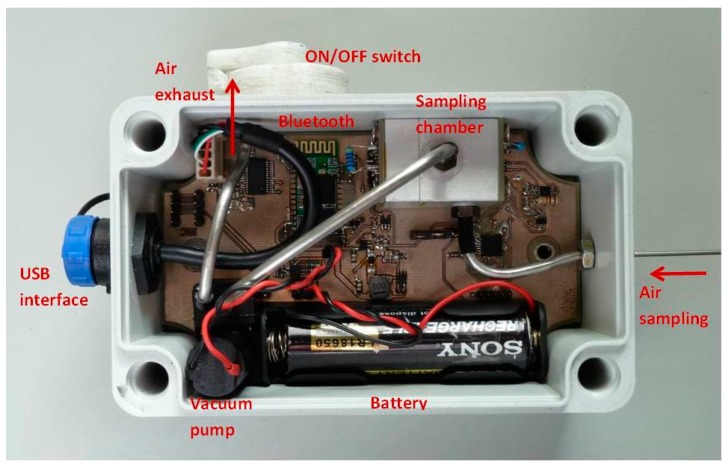
Prototype sensor device.

A portable device (Figure 2) was developed and tested for the study of the fluorescence quenching of the CP films by the nitroaromatic vapors. A Sony SLD3135 laser diode (405 nm; 50 mW) with integrated monitoring photodiode was used as excitation source and a Vishay BPW34 Silicon PIN photodiode covered with a green filter (Edmund Optics #43-934) to avoid excitation light from reaching the measuring photodiode was used for the detection. The general architecture is described elsewhere [14].

## 3. Results and Discussion

**Figure 3:**
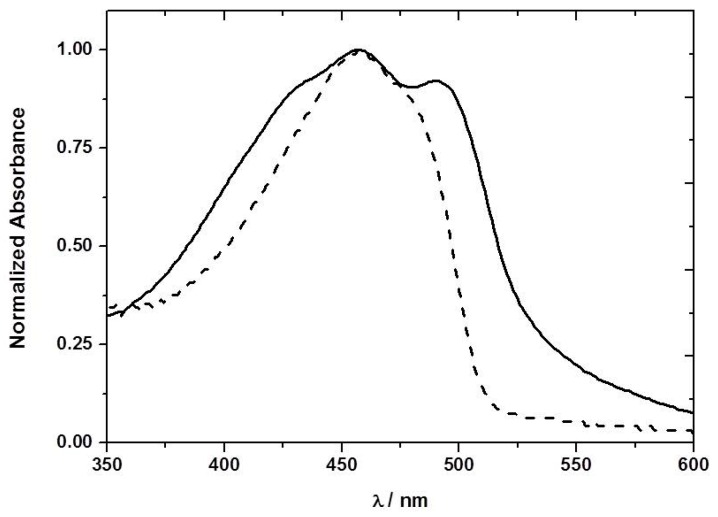
Absorption spectrum of the F8T2 ethyl cellulose film (solid line) and in toluene solution (dashed line).

**Figure 4:**
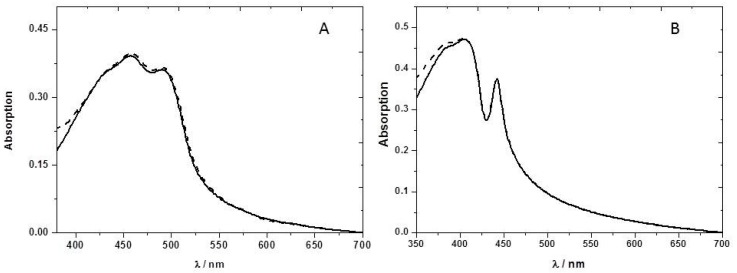
(**A**) Absorption spectra of the F8T2 film in the presence (dashed line) and absence (solid line) of nitrobenzene vapor; (**B**) Absorption spectra of the PFO film in the presence (dashed line) and absence (solid line) of nitrobenzene vapor. Both CPs are incorporated in an ethyl cellulose matrix.

The ground-state absorption spectrum of F8T2 was obtained in toluene solution and in ethyl cellulose films (Figure 3). In the case of the thin film a new band is observed at longer wavelengths (492 nm). A number of possible explanations exist for this new band. However, detailed analysis suggests that it is associated with the so-called β phase of the F8T2 [15]. The β phase is a metastable state with part of the CP in a rigid extended structure, and can be formed through evaporation of an appropriate solvent, by treatment of the film or one of the other phases by solvent vapor, or by keeping the polymer in a restricted environment. The β-phase peak was observed originally by Bradley and co-workers [16] in PFO polystyrene films and PFO solutions in poor solvents (such as methylcyclohexane) [17]. We observed this new band at 492 nm in the absorption spectrum of the PFO ethyl cellulose films (Figure 4), even though F8T2 is a less rigid polymer than PFO, strong support has been presented from steady-state and time-resolved fluorescence and fluorescence anisotropy measurements for the formation of the β-phase with this polymer [18].

The presence of NB vapor does not change significantly the absorption spectrum (Figure 4) of the PFT2 or PFO ethyl cellulose films, suggesting the absence of ground-state complexation.

**Figure 5:**
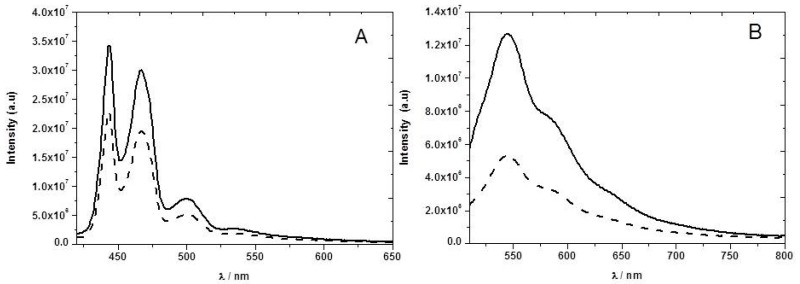
Fluorescence spectra in the absence (solid line) and presence of saturated NB vapors at room temperature (dashed line) of: (**A**) PFO in ethyl cellulose film, λ_exc_ = 410 nm, and (**B**) F8T2 in ethyl cellulose film, λ_exc_ = 490 nm.

The F8T2 emission spectrum has a maximum at 545 nm (Figure 5). In the presence of NB vapor we observed a decrease of the emission intensity of about 42%. In the case of the PFO, the emission showed a structured fluorescence spectrum between 400 and 600 nm, attributed to at least three vibronic components. We observed only a 34% drop in the fluorescence emission intensity in the presence of NB vapor at 445 nm. As mentioned, the CPs are good electron donors and their fluorescence is quenched by NB through photoinduced electron transfer. The amplifying nature of the exciton delocalization in the conjugated polymers makes them highly sensitive materials to quenching by nitroaromatic vapors.

Time-resolved fluorescence decays of CPs in thin films of ethyl cellulose recorded at the maximum emission wavelength are well fitted with sums of three exponentials (Table 1). This agrees with previous studies [18] showing triple exponential decays of F8T2 in methylcyclohexane (MCH), with lifetimes of 650 ps, 440 ps, and 20 ps. The longest decay time (650 ps) is assigned to the β-conformation and the intermediate lifetime (440 ps) to the α-conformation [18]. The shortest time (20 ps) may result from solvent/conformational relaxation or intramolecular energy transfer from non-ordered to ordered chain segments. Studies of PFO in toluene solution [19] also show a complex decay, a sum of two or even three exponentials are being required to obtain good fits. A fast component of about 20 ps is found, and is more important (with greater amplitude) at the onset of the emission band. An intermediate component is also observed around 90 ps and a predominant decay time around 360 ps is observed independent of the emission wavelength and attributed to the PFO intrinsic fluorescence lifetime. In thin films, the decay is again described by a of sum of three exponentials, however, at long wavelengths, it is dominated by a long component of 3 ns, attributed to the presence of photooxidized species, such as keto defects and other emissive defects, which are easily populated by efficient energy migration [20].

The drop in the average lifetimes (τ⁄τ_0_) of F8T2 and PFO, resulting from the presence of NB vapors, is 35% and 28%, respectively. Comparing these values with those measured in steady-state conditions, 42% and 34% for F8T2 and PFO, respectively, it is concluded that the quenching induced by the nitroaromatics is predominantly a dynamic process. This is important as it favors reversibility of the sensing system.

**Figure 6:**
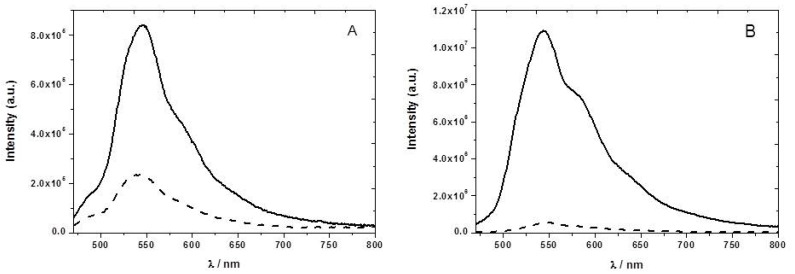
(**A**) Emission spectra of the F8T2 ethyl cellulose film with 1% (*w*/*w*) of PEG 3400 in the absence (solid line) and after three minutes of exposure to nitrobenzene (8.74 × 10^−6^ M) vapor (dashed lines); (**B**) Emission spectra of the F8T2 ethyl cellulose film with 1% (*w*/*w*) of PEG 3400 in the absence (solid line) and after three minutes of exposure to 2,3-dinitrobenzene (1.83 × 10^−5^ M) vapor (dashed lines). Excitation wavelength was 450 nm.

To increase the sensitivity towards detection of nitroaromatic vapors of the P8T2 ethyl cellulose films, we have tested the effect of adding different plasticizers to the ethyl cellulose matrix. Plasticized films show a very high sensitivity towards nitroaromatics when compared with the non-plasticized ones, probably due to higher permeability (Figure 6). For example, using only 1% (*w*/*w*) of the plasticizer PEG 3400, we obtained in a short time (three minute) of 2,3-dinotrobenzene, DNB, vapor exposure a fluorescence quenching of 95% (Figure 6B).

We can see from Figure 6 a stronger fluorescence quenching caused by the DNB vapors. This higher quenching efficiency may arise from the higher electron affinity of DNB, outweighing its lower vapor pressure [21]. In all the studied plasticized films, the sensitivity towards nitroaromatic vapors increases when compared with the neat ethyl cellulose films, as can be seen from the decrease in fluorescence lifetimes in Table 2.

By comparing the values in the lifetime attenuation for the plasticized and non-plasticized films (Table 2), we observe that the addition of only 1% by weight of plasticizer to the neat ethyl cellulose matrix increases the sensitivity of F8T2 towards these nitroaromatic vapors by ca. 5% (NB), 29%, and 35% (DNB). The molecular weight of the plasticizer does not appear to have a significant influence on the increase in the sensitivity of the CP, but it is noted that a lower molecular weight one seems to facilitate a slightly stronger quenching with both NB and DNB. This may be a result of better compatibility with the EC matrix, and, hence, production of a more amorphous structure.

**Figure 7:**
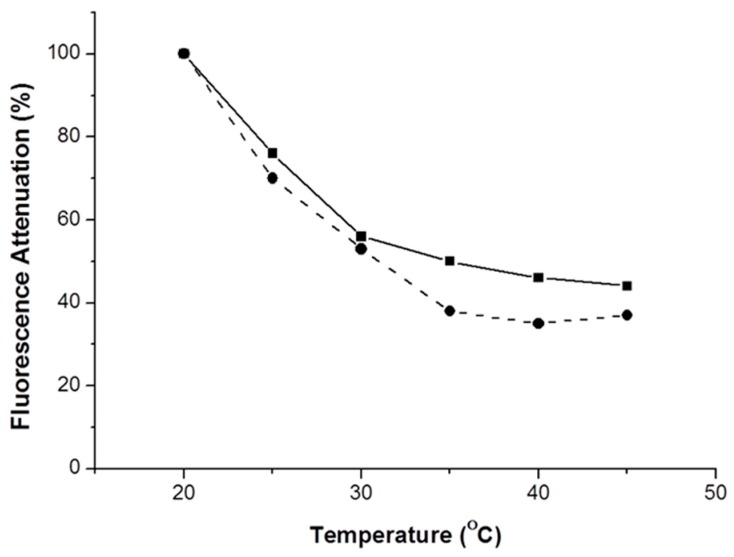
Fluorescence attenuation of the F8T2 ethyl cellulose film with 1% (*w*/*w*) of PEG 3400 in the absence (solid line) and presence (dashed line) of DNB vapor with as a function of the temperature. Excitation wavelength was 450 nm.

The thermal behavior of these plasticizers films was studied. Neat ethyl cellulose has a glass transition temperature (Tg) at 130–133 °C [22]. As expected, the addition of plasticizer decreases the Tg of the samples. For example, using 25% (*w*/*w*) PEG 400 the Tg drops to 70 °C [21]. This strong decrease means that there is a good compatibility between the polymer matrix and the plasticizer. In general, plasticizers reduce polymer interchain interactions by distributing themselves homogeneously within the polymer, hence increasing the free volume. However, a reduction in the Tg value down to near room temperature will result in an increase in chain mobility and, consequently, could enhance the crystallization of films by reducing the energy required for this process. This phenomenon would lead to structural changes resulting in loss of transparency. The thermal stability of the films was studied by monitoring the fluorescence intensity with an increase of the temperature. These experiments (Figure 7) were performed in the absence and in the presence of DNB vapors.

It can be seen that the increase of DNB vapor pressure with the temperature is not the major factor involved. Instead, the temperature dependence appears to result from the decrease in Tg upon PEG addition.

**Figure 8:**
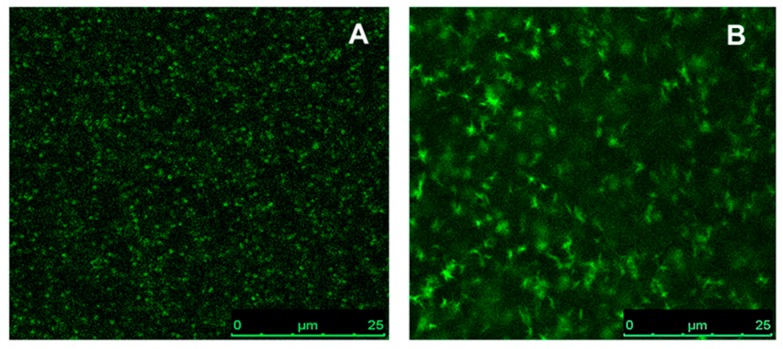
Typical images of (**A**) PFO and (**B**) F8T2 in neat ethyl cellulose film, observed by confocal microscopy. Excitation wavelength was 458 nm and emission wavelength in the range of 510–700 nm. These pictures have 512 × 512 pixels, using a pinhole of 1 AU, zoom 4×, and 400 Hz. The scale bars are 25 μm.

The morphology of the CP films was studied by confocal fluorescence microscopy. Typical images obtained from this technique are shown in Figure 8, in which the green spots represent the emission of the CPs. These films do not exhibit bulk phase separation at the magnifications studied, but some polymer aggregation can be observed, especially in the ethyl cellulose film containing F8T2.

**Figure 9:**
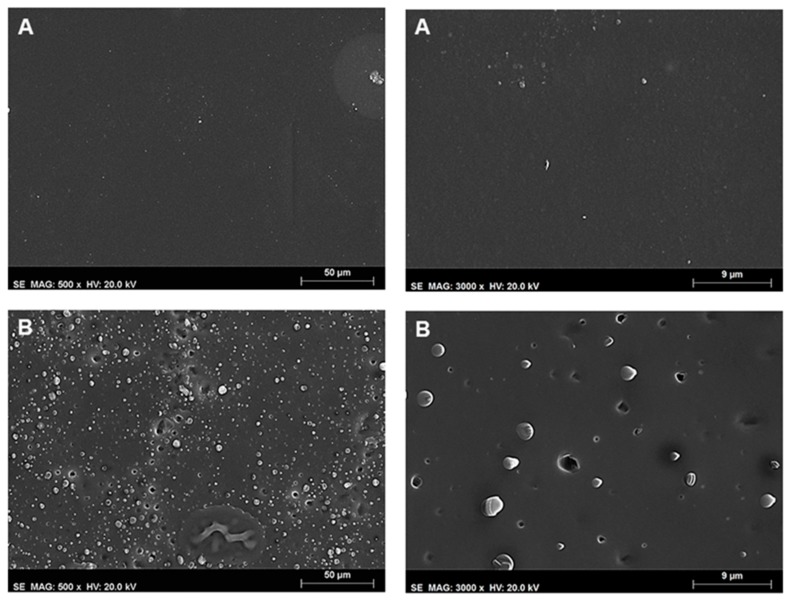
SEM images of ethyl cellulose films containing (**A**) F8T2 and (**B**) F8T2 and 1% PEG 600. The scale bars are 50 μm and 9 μm.

The surface morphology of the films was studied using scanning electron microscopy (SEM) (Figure 9). The surface of neat ethyl cellulose film (not shown) is rather smooth, compact, and featureless. However, in the case of ethyl cellulose, F8T2 blends, phase separated zones are observed for concentrations above the incorporation capacity of CPEs into ethyl cellulose films, Figure 9A. The addition of a plasticizer to the neat ethyl cellulose can be seen in Figure 9B to introduce some porosity. This film has pores with diameters between 1.5 and 3 μm, randomly distributed. The formation of these pores in the plasticized films may explain, in part, the increased sensitivity to the nitroaromatic vapors observed by fluorescence quenching of the CPs.

**Figure 10:**
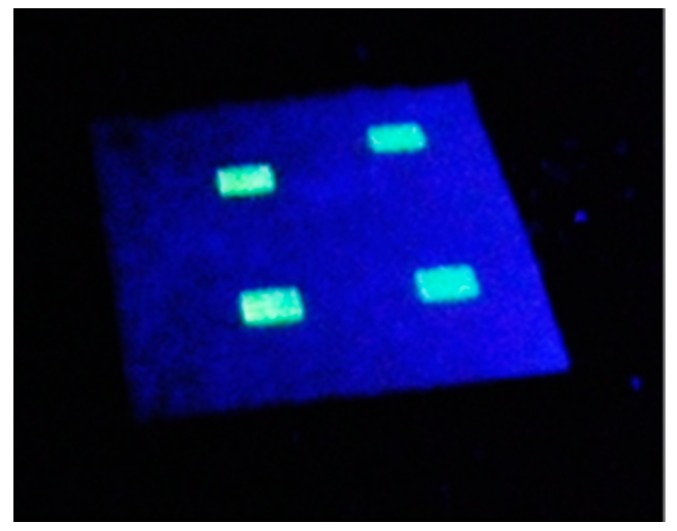
Photograph of the P8T2 IJMP imprinted film on ethylcellulose under UV light.

Fluorescence quenching based sensors normally require a reference material, such that the degree of quenching measured by the ratio of signals from sensor and reference materials. We have produced such a ratiometric system using ink-jet microprinting (IJMP) (Figure 10). The IJMP allows direct deposition of minuscule quantities of the CPs onto the ethyl cellulose film substrate (thickness 240 μm). The diameter and uniformity of the microdot can be controlled by modifying substrate surface chemistry and ink preparation [21].

We have used F8T2 as sensor material, and have incorporated another CP, F8BT (Figure 1), which is not readily oxidizable and does not exhibit any fluorescence changes in the presence of DNB and NB vapors, thereby serving as an internal reference. Table 3 shows the analysis of fluorescence decays of the imprinted ethyl cellulose sensor by IJMP. In the presence and absence of concentrated DNB vapors, we observed that F8BT did not exhibit any significant attenuation of its fluorescence lifetime, in contrast to the quenching observed with F8T2. In this case, we can use this system as a sensor with an internal reference for nitroaromatic vapors.

If we compare the values in the lifetime attenuation for the F8T2 and F8BT (Table 3), we observe that the F8T2 lifetime decreases by 52%, whereas there are insignificant changes in the F8BT lifetime. The measured sensitivity is equivalent to that obtained in plasticized ethyl cellulose films.

**Figure 11:**
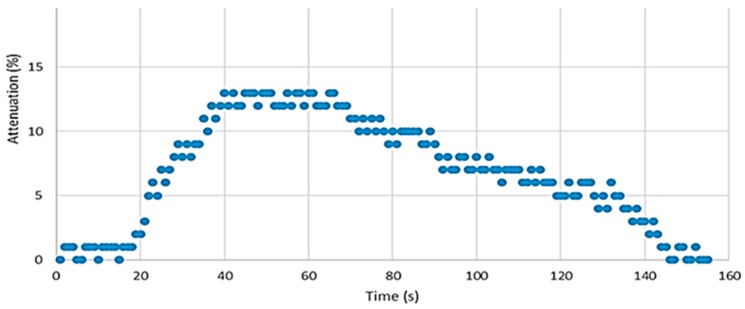
Attenuation of the fluorescence emission of the F8T2 in ethyl cellulose when exposed to NB (1.7 × 10^−5^ M) vapors for 20 s.

The sensor was tested in a dynamic setup composed of two mass flow controllers (MFCs Dwyer GFC-2102), one controlling the flow of clean air and the other controlling the flow of saturated nitrobenzene vapor, obtained from a bubbler at constant temperature and constant pressure. Both MFCs are controlled from MATLAB through a microcontroller (PIC24FV16KM202) that sets the references to the MFCs, defining the composition of the nitrobenzene-clean air mixture. As expected, the proposed differential approach guaranteed very good sensitivity and fast response from the electronics side while providing high-level input signals. Figure 11 shows the sensor response after a 20-second exposure to minute NB vapors.

For in-field applications, the fast response to the presence of a small concentration of nitroaromatics is a very positive characteristic. Although the recovery time is rather long (several minutes), this can be acceptable for scenarios where the detection of frequent changes in the analyte level is not required.

## 4. Conclusions

We have studied fluorene-based conjugated polymers as fluorescence sensing materials for nitroaromatic vapors with the overall goal of detecting explosives using such polymers. The best conjugated polymer in our studies was found to be poly(9,9-dioctylfluorenyl-2,7-diyl]-co-bithiophene] (F8T2). It is stable, has a good absorption between 400 and 450 nm, a strong and structured fluorescence around 550 nm. A 96% quenching of fluorescence, accompanied by a corresponding decrease in the fluorescence lifetime, is seen on exposure of the plasticizer film of F8T2 ethylcellulose to the model compounds nitrobenzene (NB) and 1,3-dinitrobenzene (DNB) vapors, both from the family of common explosives vapors.

Furthermore, it was demonstrated that ink-jet microprinting can be used as a convenient approach to easily and rapidly fabricate films containing these sensors and inert reference materials, with the same sensitivity of plasticized ethyl cellulose films towards nitroaromatic vapors.

A sensor prototype based on the F8T2 conjugated polymer was developed and tested. The ability of the sensor to detect small quantities of nitrobenzene was confirmed. The sensor prototype showed very fast response (a few seconds) to the presence of small concentrations of the target analyte, but also showed a large recovery time, which limits its potential applications. This slow recovery may result from the designed sampling chamber and also from the time required for desorption of the nitrobenzene molecules from the polymer surface. Both of the above aspects will be addressed in a future designs, optimizing polymer thickness and porosity, and optimizing the shape and arrangement of the sampling chamber.

## Figures and Tables

**Table 1:** Decay times and amplitudes of the PFO and F8T2 in ethyl cellulose thin film without NB vapors and in the presence of NB vapors.

	NB	$\tau_{1}$/ns ($f_{1}$ *)	$\tau_{2}$/ns ($f_{2}$ *)	$\tau_{3}$/ns ($f_{3}$ *)	$\tau_{\mathrm{average}}$/ns	$\tau_{fluor}$ change (%) **
**F8T2**	Without	0.03 (0.10)	0.43 (0.55)	0.62 (0.35)	0.40	65
	With	0.005 (0.07)	0.04 (0.51)	0.29 (0.42)	0.14	
**PFO**	Without	0.16 (0.26)	0.31 (0.63)	1.70 (0.11)	0.43	72
	With	0.05 (0.37)	0.08 (0.52)	0.55 (0.11)	0.12	

* computed from the individual lifetimes and pre-exponential factors: $\alpha_{1},\;\alpha_{2}\;\mathrm{and}\;\alpha_{3},\;f_{1}=\;\alpha_{1}\tau_{1}/(\alpha_{1}\tau_{1}+\;\alpha_{2}\tau_{2}+\;\alpha_{3}\tau_{3}$), $f_{2}=\;\alpha_{2}\tau_{2}/(\alpha_{1}\tau_{1}+\;\alpha_{2}\tau_{2}+\;\alpha_{3}\tau_{3}$) and $f_{3}=1-\left(f_{1}+f_{2}\right)$. ** computed as: ($\frac{\tau_{\mathrm{average}\;\mathrm{without}\;\mathrm{NB}\;\mathrm{vapors}}-\tau_{\mathrm{average}\;\mathrm{with}\;\mathrm{NB}\;\mathrm{vapors}}}{\tau_{\mathrm{average}\;\mathrm{without}\;\mathrm{NB}}}$) × 100.

**Table 2:** Decay times of F8T2 in ethyl cellulose thin films with different plasticizers in the presence and in the absence of nitroaromatic vapors.

	NB			DNB		
	τ_average without NB vapors_/ns	τ_average with NB vapors_/ns	$\tau_{fluor.}$ Decrease (%) *	τ_average without NB vapors_/ns	τ_average with DNB vapors_	$\tau_{fluor.}$ Decrease (%) *
**Neat Ethyl Cellulose**	400	340	**15**	400	300	**25**
**1% PEG 3400**	380	330	**13**	375	200	**47**
**1% PEG 600**	380	300	**21**	380	180	**53**

* computed as: ($\frac{\tau_{\mathrm{average}\;\mathrm{without}\;\mathrm{NB}\;\mathrm{or}\;\mathrm{DNB}\;\mathrm{vapors}}-\tau_{\mathrm{average}\;\mathrm{with}\;\mathrm{NB}\;\mathrm{or}\;\mathrm{DNB}\;\mathrm{vapors}}}{\tau_{\mathrm{average}\;\mathrm{without}\;\mathrm{NB}\;\mathrm{or}\;\mathrm{DNB}\;\mathrm{vapors}}}$) × 100.

**Table 3:** Decay times of the F8T2 and F8BT imprinted by IJMP in ethyl cellulose film in presence or absence of nitroaromatics vapors.

F8T2			F8BT		
τ_average without DNB vapors_/ns	τ_average with DNB vapors_/ns	$\tau_{fluor.}$ Decrease (%) *	τ_average without NB vapors_/ns	τ_average with DNB vapors_	$\tau_{fluor.}$ Decrease (%) *
1. 8	0.86	52	0.79	0.78	1

* computed as: ($\frac{\tau_{\mathrm{average}\;\mathrm{without}\;\mathrm{NB}\;\mathrm{vapors}}-\tau_{\mathrm{average}\;\mathrm{with}\;\mathrm{NB}\;\mathrm{vapors}}}{\tau_{\mathrm{average}\;\mathrm{without}\;\mathrm{NB}\;\mathrm{vapors}}}$) × 100.
